# Processed meat consumption and associated factors in Chile: A cross-sectional study nested in the MAUCO cohort

**DOI:** 10.3389/fpubh.2022.960997

**Published:** 2022-08-18

**Authors:** Jenny Ruedlinger, Vicente Cid-Ossandón, Andrea Huidobro, Vanessa Van De Wyngard, Claudio Vargas, Catterina Ferreccio

**Affiliations:** ^1^Facultad de Medicina, School of Medicine, Pontificia Universidad Católica de Chile, Santiago, Chile; ^2^Advanced Center for Chronic Diseases, Universidad de Chile and Pontificia Universidad Católica de Chile, Santiago, Chile; ^3^Facultad de Medicina, School of Medicine, Universidad Católica del Maule, Talca, Chile; ^4^Departamento de Matemáticas y Ciencias de la Computación, Facultad de Ciencias, Universidad de Santiago de Chile, Santiago, Chile

**Keywords:** processed meat, meat consumption, Latin American, Mediterranean diet, population-based cohort, chronic diseases, cancer

## Abstract

Processed meat consumption is increasing in Latin America. While in developed countries processed meat consumption has been associated with cardiovascular diseases and cancer, our region lacks data associated to its consumption and health impact. We characterized processed meat intake and associated factors in a population-based cohort of a Chilean agricultural county, MAUCO. We analyzed baseline dietary data of 7,841 participants, 4,358 women and 3,483 men (38–77 years), who answered an adapted Mediterranean index food frequency questionnaire. Eight percent of the participants presented high processed meat consumption (≥5 times per week). We explored associations of processed meat consumption with participant characteristics using multinomial logistic regression models. Main factors associated with higher consumption were being men, younger and currently employed, and having a high intake (>4 times per week) of red meat (Odds ratio, 2.71, 95% CI 2.10–3.48), butter/cream (1.96, 1.60–2.41), whole-fat dairy products (1.32, 1.04–1.67) and a high intake (≥1 time per day) of sugary snacks/sweets (2.49, 2.04–3.03) and sugary drinks (1.97, 1.63–2.38). Processed meat consumption associated to chronic diseases, particularly cardiovascular disease (Prevalence ratio, 2.28, 95% CI 1.58–3.29). Obesity mediated this association in a proportion of 5.0%, whereas for diabetes the proportion was 13.9%. In this population, processed meat was associated with other unhealthy dietary and lifestyle factors, as well as with chronic diseases, particularly cardiovascular disease.

## Introduction

Worldwide daily consumption of red meat (beef, lamb and pork) is greater than what was recommended by the World Cancer Research Fund (WCRF) in 2018 (350–500g of weekly intake) (1) and even more than suggested as optimal by The Global Burden of Disease Study (GBD) (18–27 g per day) (2). Although both processed meat (i.e., meat transformed through salting, curing, fermentation, smoking, or other processes in order to enhance flavor or improve preservation) and red meat (unprocessed) have been associated with chronic diseases in different populations, the risk is clearer and stronger for processed meat (2–7), which represents a popular form of meat consumption. The associated health conditions are cardiovascular diseases like stroke (7) and coronary heart disease (5), cancer like renal cell carcinoma (8), breast (9–11), gastric (12, 13) and colorectal (14, 15), and type 2 diabetes (16, 17), including mortality due to these and all-causes (3, 4, 18, 19). High processed meat consumption is also associated to overweight and obesity (19), metabolic syndrome (20, 21), and hypertension (22, 23). Moreover, the International Agency for Research on Cancer classified processed meat as “carcinogenic to humans” (Group 1) on the basis of sufficient evidence for colorectal cancer, although a positive association was also reported for stomach cancer (6). Despite recent controversies questioning the evidence behind current international recommendations of limiting red meat and processed meat consumption (24), these risks continue to be warned as the quality of the evidence improves (25, 26).

Average processed meat consumption is well above the suggested intake (2) even in low-income settings in Latin America (27) and some regions of Africa and Asia (2). Low socioeconomic status has been related to higher processed meat consumption, particularly in Chile and Argentina (28), and also in high-income countries in Europe (29). In Latin America, processed meat consumption varies between Southern, Central, Tropical and Andean regions, ranging from 6.2 g/day to 24.8 g/day (28). Among the Organization for Economic Cooperation and Development countries, Chile has one of the highest red meat intakes per year (30). This increased from 81.2 to 89.1 kg/per capita between 2008 and 2013 (31), with pork being the most consumed (31, 32). In Chilean adolescents, in particular, processed meat intake is higher than unprocessed red meat (33).

As shown, most evidence of the health effects of processed meat consumption come from Asian, European and US populations. Given the high and growing consumption of processed meat in Latin America as well as the sustained increase of the chronic diseases associated to it -reported elsewhere (2, 34–38)- an assessment of the magnitude and impact of this preventable risk factor is urgently needed in Latin America. In Chile, processed meat intake has received little attention, even though previous studies showed the country had the highest consumption among 8 Latin American countries (28).

The MAUCO Cohort is located in the agricultural Molina County in the Maule Region, 200 km south of the capital city of Santiago. This population is characterized by the fact that in recent decades it has gone from being undernourished to suffering from excess caloric intake, and has one of the highest national rates of cardiovascular disease, stomach cancer and gallbladder cancer. In addition, poverty rates here dropped significantly in a short period of time (2009–2011) which implied advances in terms of sanitation. As the county economy is agriculture based, pesticide exposure is of particular interest in this population, as well as the study of other environmental risk factors in the development of chronic diseases (39). Here we present the frequency of processed meat consumption and its associated sociodemographic, health and lifestyle factors in this population-based cohort.

## Materials and methods

### Study design and setting

We conducted a cross-sectional analysis of baseline dietary data of all participants enrolled in the Maule cohort (MAUCO), from the agricultural county of Molina in the Maule Region, central Chile (39). This region is characterized for presenting one of the highest incidence rates for gastric cancer in men and women per 100,000 (regional 46.3 and 17.7 vs. 34.1 and 12.8 nationwide, respectively) (40), one of the highest mortality rates for colon cancer per 100,000 (regional 8.6 vs. 7.19 nationwide) (41) and a prevalence of cardiovascular risk factors above national average (42). MAUCO seeks to analyze the natural history of chronic diseases in Chile. Details of cohort recruitment and study protocols have been described elsewhere (39). In brief, selection criteria were: to be a resident of Molina for at least 6 months and without plans to move for the next 3 years, aged 38 to 74 years, and being able to consent autonomously. Individuals with a diagnosis of terminal illness were excluded (43). We included the 7,841 participants enrolled in the cohort between December 2014 and December 2019 and who had answered the question on consumption of processed meat. Written informed consent was obtained from all participants.

### Dietary assessment

Baseline dietary assessment of the regularly consumed foods in the last 12 months was based on a food frequency questionnaire which included items from a Mediterranean diet survey (44). This Mediterranean diet was designed based on traditional food consumption habits in the European Mediterranean region with modifications to incorporate Chilean dietary habits. This “Chilean Mediterranean Diet Index” (Chilean MDI) was the first Mediterranean diet quality index to be adapted and validated specifically for use in Chile (45). The MAUCO food frequency questionnaire, adapted from the Chilean MDI, was applied in person by trained field staff. For each item, four to six consumption frequencies were available, depending on the food item. For example, response options ranged from “none” to “>3 time per day” for vegetables and to “>8 times per week” for whole-fat dairy products. With the exception of processed meat, the consumption frequency of all other dietary items was categorized into two levels based on recommendations for the Mediterranean diet (44–46). Box 1 summarizes the foods and cut-points used.

Box 1 **Dietary items in food frequency questionnaire and cut-off points used.**

***I. Items categorized according to frequency of intake***
**1 Vegetables** (1 serving = 1 cup) raw or cooked, consumed as salads, stews, soups made of natural vegetables, and hot side dishes (servings per day: ≥1/ <1).
**2 Fruits** (1 serving = 1 cup), including raw, cooked or dried fruits as dried peaches, raisins, dried figs, others (servings per day: >2/ ≤ 2).
**3 Legume** (1 serving = 1 plate), as soups, stews and salads, including lentils, chickpeas, beans, and dried or dehydrated peas (servings per week: >2/ ≤ 2).
**4 Nuts** (1 serving = 1 handful) such as walnuts, almonds, hazelnuts, cashews, pistachios, peanuts, seeds, etc. (servings per week: >2/ ≤ 2).
**5 Whole grain cereals** (1 serving = 1 cup or 2 slices of bread or 6 cookies/crackers), considering brown rice and whole-wheat pasta, whole-wheat bread, whole-grain breakfast cereals, whole-wheat cookies or crackers, and all kinds of dough or dishes made with whole-grain cereals (servings per day: ≥2/ <2).
**6 White meat** including poultry, chicken, turkey and lean pork (times per week: ≤ 4/>4).
**7 Red meat** considering beef, lamb and fatty pork (times per week: ≤ 4/>4).
**8 Fish or seafood** (times per week: >2/ ≤ 2).
**9 Skimmed/fermented dairy products** including skimmed, low-fat or fermented milk, all kind of yogurt, cultured milk, cottage cheese and fresh cheese (times per week: >4/ ≤ 4).
**10 Whole-fat dairy products** such as milk and yogurt (times per week: ≤ 4/>4).
**11 Butter or cream** (times per week: ≤ 4/>4).
**12 Olive oil** (teaspoons per day: ≥3/ <3).
**13 Avocados** (units per week: >3/ ≤ 3).
**14 Sugary snacks/sweets** including candies, cookies, chocolates, and desserts with sugar like jelly, pies, cakes (times per day: <1/≥1).
**15 Sugary drinks** (times per day: <1/≥1).
**16 Sugar** (teaspoons per day: <4/≥4).
**17 Fried foods** (times per week: ≤ 1/>1).
**18 Fresh green chili pepper** (times per week: <5/≥5).
**19 Fresh red chili pepper** (times per week: <5/≥5).
**20 Dried red chili pepper** such as chili powder, chili paste or Chilean smoked chili pepper known as merken (tablespoons per week: <5/≥5).
***II. Processed Meat*** Meat products transformed through salting, curing, fermentation, smoking, or other processes, e.g., bacon, ham, sausages (including Chilean products as paté, longanizas and other meat by-products) and pre-made hamburgers. Participants were categorized into five groups (weekly frequency: non-consumers, <1, 1, 2–4 and ≥5 times per week).

### Sociodemographic, lifestyle, anthropometric and health variables

All participants answered surveys about sociodemographics, lifestyle (i.e., tobacco and alcohol consumption), personal and family medical history, health status, and employment history, among others. Participants provided fasting blood, and received a hepatobiliary ultrasound exam, anthropometry and other physical (blood pressure, tooth count) and laboratory tests (glycemia, triglycerides, cholesterol, alanine aminotransferase, aspartate aminotransferase, among others). Metabolic syndrome score was constructed considering abdominal obesity, high Triglycerides, low HDL cholesterol, high blood pressure and high fasting glucose, according to ATP III criteria (47).

For the present analysis, the following variables were included: sex (male, female), age (years, 38–74), schooling (years completed); self-identified ethnicity (Chilean/Latin, other nationalities or ethnic groups); health insurance (public, private/other); employment status (occupied/employed, not employed); smoking status (current, former/never); drinking pattern (binge drinking: ≥3 drinks for women or ≥4 drinks for men per occasion; abstainers/other drinking patterns); number of chronic conditions (≥2 or <2), including diabetes (self-report or fasting glycemia ≥126 mg/dl or use of hypoglycemic drugs), cardiovascular disease (self-reported history of heart disease, heart failure, stroke or other, excluding hypertension), cancer (self-reported history), digestive symptoms (biliary colic, gastroesophageal reflux and gastritis symptoms in the last 12 months), non-infectious digestive diseases (self-reported history of gastric ulcer, irritable bowel syndrome, inflammatory bowel disease or other) and hypertension (use of hypotensive drugs or measured systolic blood pressure ≥130 mm Hg or diastolic blood pressure ≥80 mm Hg); remaining teeth (<20 or ≥20); waist circumference (cm); body mass index (BMI: >30kg/m2; ≤ 30kg/m2); Ultrasound-detected (48) fatty liver (any degree: yes; no) and gallbladder disease including cholecystectomy or gallstones (yes; no); metabolic syndrome (47) (yes; no); fasting blood glucose ≥126 mg/dl (yes; no); Low-Density Lipoprotein cholesterol (LDL) >160 mg/dL (yes; no); triglycerides ≥200 mg/dL (yes; no); High-Density Lipoprotein cholesterol (HDL) ≤ 40 mg/dL in men, ≤ 50 mg/dL in woman (yes; no); Aspartate Aminotransferase (AST) >48 UI/L (yes; no); and Alanine Aminotransferase (ALT) >55 UI/L (yes; no).

### Statistical analysis

To characterize processed meat consumption we analyzed baseline sociodemographic, lifestyle and health characteristics of participants across categories of intake. We present this data as prevalence for categorical variables or as mean ± standard deviation (SD) for continuous variables; reporting p**-**Values for trend. We obtained odds ratios (OR) and 95% confidence intervals (CI) by logistic regression using processed meat consumption as the explained variable dichotomized into <1 time per week (reference) vs. ≥1 times per week, adjusted by age, sex and schooling.

We also conducted multinomial logistic regression models to explore associations between sociodemographic, lifestyle and dietary variables with processed meat consumption as the outcome variable in four levels (<1 time per week as reference; 1 time per week; 2–4 times per week; and ≥5 times per week). In order to keep most participants in this analysis, we conducted multiple imputation of missing values using MICE (Multiple Imputation by Chained Equations). We created 100 imputed databases. To impute the missing values of each variable, we specified a predictive mean matching model using the 27 variables described. In each imputed dataset we performed a stepwise procedure with backward/forward direction to determine the best multinomial model to explain the outcome (processed meat consumption frequency). According to Akaike information criterion, where the variables that remain in the final model are registered in each of the 100 databases. for the final model, we considered the variables that remained in at least 60% of the models through stepwise (Supplementary Table S1). Finally, five imputed databases were created with MICE and a multinomial model was fitted with the selected variables; all the results of the analysis were aggregated with rubin's rule applying the corresponding transformations (49). We use this method under the assumption that the missing observations of the covariates are missing at random (MAR). We explore this by assessing the relationship between variables and missingness for each variable using the chi-square or kruskall wallis test, as appropriate. Given the relationships we observed (see Supplementary Table S2) and since in epidemiological research missingness appears to be typically MAR (50). We consider our assumption to be feasible. Results were expressed as OR. All models were adjusted by sex, age, schooling, employment status and red meat consumption.

To better understand whether the association between chronic diseases and processed meat consumption is influenced by the presence of obesity, we explored separately the different chronic conditions in the subgroups of high and low processed meat consumers with and without obesity. We reported prevalence ratio adjusted by age, sex, schooling, smoking and binge drinking using logistic regression. To further confirm if obesity was mediating these associations, we run a mediation analysis in the same subgroup of participants. We reported Average Causal Mediation Effect (ACME), Average Direct Effect (ADE) and proportion mediated, all estimated with R using package 'mediation' with non-parametric bootstrap.

Analyses for the multiple imputation routine were also performed in R 4.0.3 and MICE Package 3.13.0. All other data analyses were performed using stata (StataCorp. 2019. Stata Statistical Software: Release 16. StataCorp LLC, College Station, TX, USA). The level of significance of each risk estimate was set at 0.05.

## Results

### Participants

The study sample included 7,841 MAUCO participants with information about processed meat consumption at baseline; 55% women, a mean age of 53.5 ± 9.7 years, with 8.8 ± 4 years of schooling.

### Sociodemographic, lifestyle, health and dietary characteristics in relation to processed meat consumption

The proportion of participants with missing data are reported in Supplementary Table S3. A high intake of processed meat (≥5 times per week) was reported by 8% of the participants (7% of women and 9% of men); 33% reported non-consumption, 21% reported <1 time per week, 19.2% 1 time per week and 18.6% 2–4 times per week. Table 1 shows the prevalence (adjusted by age, sex and schooling) of sociodemographic, lifestyle, and health characteristics of MAUCO participants according to their distribution across the five processed meat consumption categories. Participants who ate processed meat more frequently tended to be male, younger, currently employed and with a greater proportion of smokers and binge drinkers. Regarding health conditions, those who ate processed meat more frequently were more likely to be obese and to have **two** or more chronic conditions, fatty liver, metabolic syndrome and elevated levels of fasting blood glucose, triglycerides and ALT enzyme. Some of these associations were also evident in the logistic model with processed meat consumption as a dichotomized variable (<1/≥1 per times per week), presented in Table 2.

**Table 1:** Profile of participants by weekly frequency of processed meat consumption.

			**Frequency of processed meat consumption**					
	**Baseline characteristics**	**Overall**	**None** ***n* = 2,596 (33%)**	** <1/week** ***n* = 1,651 (21%)**	**1/week** ***n* = 1,504 (19%)**	**2–4/week** ***n* = 1,459 (19%)**	**≥5/week** ***n* = 631 (8%)**	***P* trend^z^**
**Sociodemographics**								
Sex	Men	44.4	33.3	46.0	48.1	55.6	51.3	<0.0001
Age	Years, mean±SD	53.5 ± 9.7	55.3 ± 9.4	54.2 ± 9.6	52.5 ± 9.6	51.5 ± 9.6	51.9 ± 9.9	<0.0001
	Years, ≤ 55	58.9	50.9	57.1	63.4	68.0	65.5	<0.0001
Ethnicity	Chilean/Latin	97.0	97.0	96.8	97.3	96.8	97.3	0.77
Schooling	Years, mean±SD	8.8 ± 4.0	8.6 ± 4.1	8.9 ± 4.1	8.9 ± 4.0	8.9 ± 3.8	8.9 ± 3.8	0.65
Health insurance	Public	85.5	84.2	84.3	85.9	88.6	85.4	0.05
**Lifestyle**								
Work	Occupied/employed	80.8	78.6	79.5	81.3	81.0	91.6	<0.0001
Tobacco	Current smoker	29.6	28.2	29.0	28.8	29.8	34.8	0.0019
Alcohol	Binge drinking^a^	19.4	16.2	19.3	20.3	21.0	21.9	0.0004
**Health**								
Chronic diseases	≥2^b^	37.4	37.9	35.0	38.6	39.0	45.7	0.0022
Teeth	Remaining teeth <20	44.0	46.5	45.4	46.8	45.9	48.9	0.2
Anthropometry	Waist circumference, cm	98.9 ± 11.05	98.3 ± 11.2	98.6 ± 11.2	99.0 ± 10.9	99.6 ± 10.8	100.3 ± 10.8	<0.0001
	Body mass index >30 kg/m^2^	39.9	37.8	38.5	42.3	41.4	44.8	0.0017
Ultrasound exam	Fatty liver (any degree)	48.4	45.0	47.7	50.1	50.9	53.9	<0.0001
	Gallbladder disease^c^	32.3	32.6	32.2	31.3	33.0	31.3	0.68
Laboratory tests	Fasting blood glucose ≥126 mg/dL	8.5	8.5	7.5	7.9	9.5	10.5	0.04
	LDL >160 mg/dL	9.9	9.7	11.2	9.4	9.6	9.1	0.34
	Triglycerides ≥200 mg/dL	25.1	24.9	24.5	23.3	26.0	30.0	0.0078
	HDL ≤ 40 mg/dL or ≤ 50 mg/dL^d^	52.6	52.9	52.6	51.3	53.3	52.5	0.9
	AST >48 UI/L	6.1	4.9	6.6	5.5	7.7	6.2	0.14
	ALT >55 UI/L	10.6	9.5	10.6	10.9	11.4	12.5	0.03
	Metabolic syndrome^e^	48.5	48.8	47.4	46.2	48.9	54.3	0.0138

*Analysis in 7,841 MAUCO participants. Values are presented as percentages unless otherwise indicated. Prevalence estimated by logistic regression model. For continuous variables multiple linear regression. Adjusted by age, schooling and sex. Missing data were excluded (see Supplementary Table S3);*

a *≥3 drinks for women or ≥4 drinks for men per occasion;*

b *number of chronic conditions, including diabetes, cardiovascular disease, cancer, digestive symptoms, non-infectious digestive diseases and hypertension;*

c *Including gallstones and cholecystectomy;*

d *≤ 50 mg/dL in women, ≤ 40 mg/dL in men;*

e *≥3 of the following: abdominal obesity, high triglycerides, low HDL cholesterol, high blood pressure and high fasting glucose; LDL, low-density lipoprotein cholesterol; HDL, high-density lipoprotein cholesterol; AST, Aspartate aminotransferase, ALT, Alanine Aminotransferase;*

z *p for trend according to logistic regression model*.

**Table 2:** Sociodemographic and health factors associated with processed meat consumption at least once a week.

	**Baseline characteristic (*n*)**	**PMC ≥1/week (%)** ***n* = 3,594**	**Odds ratio (95% CI)^†^**
Sex	Men (3,483)	53.3	1.82 (1.66–2.0)
	Women (4,358)	39.8	1
Health insurance	Public (6,450)	46.5	1.24 (1.08–1.42)
	Other (1,095)	42.8	1
Employment	Occupied/employed (5,046)	49.7	1.35 (1.16–1.56)
	Not employed (1,197)	32.1	1
Alcohol intake	Binge drinking^a^ (1,520)	56.5	1.26 (1.12–1.43)
	Abstainer or another drinking pattern (6,313)	43.3	1
Chronic diseases	≥2^b^(2,102)	40.0	1.14 (1.01–1.28)
	<2 or none (3,433)	41.8	1
Body mass index	>30 kg/m^2^ (2,970)	47.1	1.19 (1.08–1.31)
	≤ 30 kg/m^2^ (4,457)	43.7	1
Ultrasound fatty liver	Yes (any degree) (3,579)	47.2	1.22 (1.11–1.34)
	No (3,823)	42.9	1
ALT	>55 UI/L (788)	53.0	1.17 (1.00–1.36)
	≤ 55 UI/L (6,607)	44.1	1

Analysis in 7,841 MAUCO participants. *

† *Odds ratios and 95% confidence intervals obtained by logistic regression using processed meat consumption (PMC) as the explained variable dichotomized into <1/week (reference, including non-consumers) vs. ≥1 times per week. Age, sex and schooling-adjusted.; Missing data were excluded (see Supplementary Table S3);*

a *≥3 drinks for women or ≥4 drinks for men per occasion;*

b *number of chronic conditions, including diabetes, cardiovascular disease, cancer, digestive symptoms, non-infectious digestive diseases and hypertension; ALT, Alanine Aminotransferase*.

Table 3 shows that participants who ate processed meat more frequently were also more likely to have a higher intake of other foods, such as red meat, butter or cream, sugary snacks and sweets, sugary drinks, refined sugar and fried foods, and a lower intake of vegetables. Consumption of a variety of chili peppers was also associated with processed meat intake. Some of these foods were also associated in the logistic model with processed meat consumption as a dichotomized variable (<1/≥1 times per week), presented in Table 4.

**Table 3:** Intake of other foods with increasing consumption of processed meat.

		**Frequency of processed meat consumption**					
**Food item** (intake category)^*^	**Overall**	**None** ***n* = 2,596 (33%)**	** <1/week** ***n* = 1,651 (21%)**	**1/week** ***n* = 1,504 (19%)**	**2–4/week** ***n* = 1,459 (19%)**	**≥5/week** ***n* = 631 (8%)**	***P* trend^z^**
Vegetables <1 time/day	34.1	31.2	34.6	33.3	33.6	36.2	0.009
Fruits ≤ 2 servings/day	95.3	94.0	95.7	96.2	95.6	96.0	0.11
Legumes ≤ 2 servings/week	82.3	84.6	84.8	84.0	80.8	68.6	<0.0001
Nuts ≤ 2 servings/week	89.3	87.7	89.8	91.6	89.6	85.6	0.19
Whole grain cereals <2 servings/day	98.3	98.4	98.9	98.5	97.8	97.2	0.008
White meat >4 times/week	4.1	4.5	2.9	3.2	3.8	8.3	0.0002
Red meat >4 times/week	11.7	8.7	7.7	10.5	16.2	27.5	<0.0001
Fish or seafood ≤ 2 times/week	94.5	93.9	95.9	96.1	94.5	88.0	<0.0001
Skimmed/fermented dairy products ≤ 4 times/week	81.4	80.0	82.4	83.2	81.4	76.9	0.08
Whole-fat dairy products >4 times/week	15.3	14.7	14.0	15.3	14.6	21.2	0.0004
Butter or cream >4 times/week	21.4	15.9	19.6	22.6	26.2	34.2	<0.0001
Olive oil <3 teaspoons/day	99.3	99.3	99.4	99.5	99.2	99.6	0.48
Avocados ≤ 3 units/week	86.0	85.4	87.3	88.4	85.3	79.1	0.0003
Sugary snacks/sweets ≥1 time/day	22.6	21.5	17.1	19.2	24.7	45.3	<0.0001
Sugary drinks ≥1 time/day	35.5	28.7	28.6	39.5	42.5	49.0	<0.0001
Sugar ≥4 teaspoons/day	38.4	35.7	37.0	38.0	41.9	42.2	0.0003
FGC pepper ≥5 times/week	7.8	6.2	5.7	6.8	6.6	23.5	<0.0001
FRC pepper ≥5 times/week	5.1	3.9	3.1	4.9	4.3	14.3	<0.0001
DRC pepper ≥5 tablespoons/week	6.9	5.4	4.4	6.4	6.9	18.8	<0.0001
Fried foods >1 time/week	20.7	16.5	20.0	21.7	25.8	22.0	<0.0001

*Analysis in 7,841 MAUCO participants. Values are presented as percentages. Prevalence estimated by logistic regression model, adjusted by age, schooling and sex; Missing data were excluded (see Supplementary Table S3);*

* *Only one category is presented; FGC, Fresh green chili; FRC, Fresh red chili; DRC, dried red chili*.

z *p for trend according to logistic regression model*.

**Table 4:** Frequency of intake of other foods associated with processed meat consumption at least once a week.

**Foods items: [reference]**	**PMC ≥1/week (%) *n* = 3,594**	**Odds ratio (95% CI)^†^** ** Ref: <1/week**
Vegetables, servings/day <1 [≥1]	49.6	1.12 (1.01–1.24)
Fruits, servings/day ≤ 2 [>2]	46.2	1.28 (1.03–1.61)
Legumes, servings/week ≤ 2 [>2]	44.4	0.73 (0.64–0.82)
Red meat, times/week >4 [≤ 4]	62.3	2.05 (1.75–2.38)
Butter or cream, times/week >4 [≤ 4]	55.2	1.69 (1.50–1.90)
Sugary snacks/sweets, times/day ≥1 [<1]	53.0	1.43 (1.28–1.60)
Sugary drinks, times/day ≥1 [<1]	58.5	1.87 (1.69–2.07)
Sugar, teaspoons/day ≥4 [<4]	49.7	1.19 (1.08–1.31)
FGC pepper, times/week ≥5 [<5]	56.6	1.65 (1.38–1.97)
FRC pepper, times/week ≥5 [<5]	60.4	1.76 (1.41–2.21)
DRC pepper, servings/week ≥5 [<5]	60.6	1.80 (1.49–2.18)
Fried foods, times/week >1 [≤ 1]	56.9	1.42 (1.26–1.60)

*Analysis in 7,841 MAUCO participants*.

† *Odds ratios and 95% confidence intervals obtained by logistic regression using processed meat consumption (PMC) as the explained variable dichotomized in <1/week (reference, including non-consumers) vs. ≥1 times per week. Age, sex and schooling-adjusted. Missing data were excluded (see Supplementary Table S3). FGC, Fresh green chili; FRC, Fresh red chili; DRC, dried red chili*.

In the multinomial logistic regression model with processed meat consumption as the outcome (4 frequency levels), male sex, lower age and being currently occupied or employed were independently associated with higher consumption. Among dietary options, high processed meat was associated with red meat, whole-fat dairy products, butter or cream, sugary snacks or sweets and sugary drinks (Table 5). On the other hand, a low intake of legumes, fish or seafood and avocados showed an inverse association to processed meat consumption. Other variables like binge drinking, fried foods and low intake of vegetables were associated to processed meat consumption but did not show a clear positive trend.

**Table 5:** Sociodemographic and diet factors associated with processed meat consumption.

	**Processed meat consumption**		
	**Odds Ratio (95% CI)^†^**		
**Variables [reference]**	**1/week**	**2–4/week**	**≥5/week**
Male [female]	1.30 (1.13–1.49)	1.72 (1.49–1.98)	1.37 (1.11–1.69)
Age (years)	0.98 (0.97–0.99)	0.97 (0.96–0.98)	0.98 (0.97–0.99)
Public health insurance [other]	1.16 (0.97–1.38)	1.44 (1.19–1.74)	1.09 (0.84–1.41)
Occupied/employed [not employed]	1.16 (0.94–1.45)	1.08 (0.89–1.32)	1.91 (1.28–2.83)
Binge drinking^a^ [abstainer or other drinking pattern]	1.19 (1.02–1.39)	1.23 (1.05–1.43)	1.19 (0.95–1.49)
Vegetables, servings/day <1 [≥1]	1.00 (0.88–1.14)	1.16 (1.02–1.33)	1.22 (1.00–1.48)
Legumes, servings/week ≤ 2 [>2]	0.94 (0.80–1.12)	0.77 (0.65–0.90)	0.50 (0.41–0.62)
Nuts, servings/week ≤ 2 [>2]	1.33 (1.07–1.65)	1.16 (0.94–1.44)	1.25 (0.93–1.69)
Red meat, times/week >4 [≤ 4]	1.26 (1.01–1.57)	1.83 (1.51–2.21)	2.71 (2.10–3.48)
Fish or seafood, times/week ≤ 2 [>2]	1.35 (0.99–1.84)	0.99 (0.74–1.30)	0.60 (0.43–0.83)
Whole-fat dairy products, times/week >4 [≤ 4]	1.04 (0.88–1.23)	0.96 (0.80–1.15)	1.32 (1.04–1.67)
Butter or cream, times/week >4 [≤ 4]	1.37 (1.17–1.59)	1.56 (1.34–1.81)	1.96 (1.60–2.41)
Sugary snacks/sweets, times/day <1 [≥1]	0.91 (0.78–1.06)	1.17 (1.00–1.35)	2.49 (2.04–3.03)
Sugary drinks, times/day <1 [≥1]	1.64 (1.44–1.87)	1.72 (1.50–1.96)	1.97 (1.63–2.38)
FGC pepper, times/week <5 [≥5]	1.00 (0.77–1.30)	0.93 (0.71–1.23)	2.42 (1.82–3.23)
DRC pepper, tablespoons/week <5 [≥5]	1.22 (0.93–1.59)	1.25 (0.96–1.66)	2.52 (1.87–3.39)
Fried foods, times/week ≤ 1 [>1]	1.20 (1.03–1.40)	1.46 (1.26–1.70)	1.13 (0.90–1.40)

*Multinomial model among 7,841 MAUCO participants; data imputed with MICE (Multiple Imputation by Chained Equations);*

† *Reference category was <1 time per week, including non-consumers;*

a *≥3 drinks for women or ≥4 drinks for men per occasion; FGC, Fresh green chili; FRC, Fresh red chili; DRC, dried red chili*.

Table 6A shows the prevalence of self-reported chronic conditions among participants divided into four groups of high and low processed meat consumers with and without obesity. As expected, hypertension and diabetes were more prevalent in obese participants among both high consumers and low consumers; similarly, cardiovascular disease and digestive symptoms were more prevalent in the obese among low consumers. However, when comparing high consumers vs. low consumers within the same obesity group, the prevalence of cardiovascular disease was 2-fold higher in high consumers vs. low consumers, in both the obese group and in the non-obese group. To further confirm if obesity was mediating these effects and to what degree, we performed a mediation analysis in the same subgroup of participants. Table 6B shows that association between high processed meat consumption and diabetes was partly mediated through obesity in a proportion of 13.9%. This mediation was also evident for cardiovascular disease and digestive symptoms, but to a lesser degree (proportions of 5.0 and 3.0%, respectively).

**Table 6A:** Association of chronic diseases and processed meat consumption by obesity status in MAUCO participants.

	**A**	**B**	**C**	**D**	**Prevalence Ratio (95% CI)** ^**†**^			
	**High consumers with obesity** ***n* = 252**	**High consumers without obesity** ***n* = 317**	**Low consumers with obesity** ***n* = 1,572**	**Low consumers without obesity** ***n* = 2,508**	**A/B Obesity effect in high PMC**	**A/C** ** PMC effect** ** in obese**	**C/D Obesity effect in low PMC**	**B/D** ** PMC effect in non-obese**
Age, years, mean±SD	51.8 ± 10.0	52.5 ± 9.9	55.1 ± 9.4	54.7 ± 9.5				
Sex, women	52.8	46.1	65.1	60.1				
Schooling, years, mean±SD	8.7 ± 3.9	9.0 ± 3.7	8.2 ± 4.2	9.0 ± 4.1				
Current smoker	34.5	38.3	23.7	29.1				
Binge drinking^a^	21.4	26.8	15.5	15.8				
Hypertension^b^ (*n* = 2,457)	58.6	43.0	62.5	47.9	2.09	1.05	1.87	0.90
					(1.47–3.00)	(0.80–1.39)	(1.63–2.15)	(0.70–1.16)
Diabetes^c^ (*n* = 735)	21.4	13.3	20.6	12.6	1.91	1.30	1.75	1.22
					(1.20–3.03)	(0.94–1.80)	(1.47–2.08)	(0.85–1.75)
Cancer^d^ (*n* = 178)	3.6	2.5	3.8	4.1	1.50	1.22	0.87	0.68
					(0.57–3.99)	(0.59–2.52)	(0.62–1.21)	(0.30–1.47)
Cardiovascular disease^e^ (*n* = 298)	15.5	12.7	10.1	6.3	1.40	2.03	1.58	2.42
					(0.69–2.79)	(1.19–3.45)	(1.22–2.05)	(1.45–4.02)
Digestive symptoms^f^ (*n* = 1,912)	46.0	46.3	45.0	38.4	0.99	1.02	1.06	1.12
					(0.95–1.03)	(0.96–1.08)	(1.01–1.10)	(1.03–1.20)
Non-infectious digestive diseases^g^	11.1	11.4	11.6	14.1	0.93	1.03	0.82	0.88
(*n* = 599)					(0.60–1.45)	(0.68–1.54)	(0.68–0.99)	(0.62–1.24)

*Data presented as percent prevalence unless otherwise specified. Prevalences are unadjusted*.

† *Prevalence ratio and 95% confidence intervals obtained by logistic regression are adjusted by age, sex, schooling, smoking and binge drinking. Obesity, body mass index >30 kg/m2;*

a *≥3 drinks for women or ≥4 drinks for men per occasion;*

b *use of hypotensive drugs or measured systolic blood pressure ≥130 mm Hg or diastolic blood pressure ≥80 mm Hg;*

c *self-report or glycemia ≥126 mg/dL or use of hypoglycemic drugs;*

d *self-reported;*

e *self-reported: history considering heart disease, heart failure, stroke or other, and excluding hypertension;*

f *biliary colic, gastroesophageal reflux and gastritis symptoms;*

g *gastric ulcer, irritable bowel syndrome, inflammatory bowel disease or other. PMC, Processed meat consumption*.

**Table 6B:** Mediation analysis of the relationship between processed meat consumption and chronic diseases using obesity as a mediator.

	**Prevalence ratio (95% CI)** ^**†**^				
	PMC → CD^*^	PMC → CD^*^	**ACME**	**ADE**	**Proportion mediated by obesity**
	**Obesity not in model**	**Obesity in model**			
Hypertension^a^ (*n* = 2,457)	1.02 (0.85–1.23)				
Diabetes^b^ (*n* = 735)	1.32 (1.04–1.68)	1.28 (1.00–1.63)	0.00541	0.03348	13.9% (3.0–57.0%)
Cancer^c^ (*n* = 178)	0.89 (0.53–1.51)				
Cardiovascular disease^d^ (n= 298)	2.28 (1.58–3.29)	2.22 (1.54–3.21)	0.00399	0.07661	5.0% (0.8–12.0%)
Digestive symptoms^e^ (*n* = 1,912)	1.07 (1.02–1.12)	1.07 (1.02–1.13)	0.00220	0.07000	3.0% (0.7–13.0%)
Non-infectious digestive diseases^f^ (*n* = 599)	0.93 (0.71–1.21)				

*Obesity, Body mass index >30 kg/m2*.

† *Prevalence ratio and 95% confidence intervals obtained by logistic regression are adjusted by age, sex, schooling, smoking and binge drinking*.

* *Prevalence ratio for CD between high PMC and low PMC;*

a *use of hypotensive drugs or measured systolic blood pressure ≥130 mm Hg or diastolic blood pressure ≥80 mm Hg;*

b *self-report or glycemia ≥126 mg/dL or use of hypoglycemic drugs;*

c *self-reported;*

d *self-reported: history considering heart disease, heart failure, stroke or other, and excluding hypertension;*

e *biliary colic, gastroesophageal reflux and gastritis symptoms;*

f *gastric ulcer, irritable bowel syndrome, inflammatory bowel disease or other. PMC, Processed meat consumption; CD, Chronic disease; ACME, Average causal mediation effect; ADE, Average direct effect*.

## Discussion

A more frequent consumption of processed meat was associated with male sex, younger age, being employed, binge drinking, a higher consumption frequency of red meat, butter or cream, sugary snacks/sweets, sugary drinks, fried foods, legumes and fish or seafood, and a low intake of vegetables. Participants with higher processed meat consumption were also more likely to be obese and to have multiple chronic conditions, fatty liver, metabolic syndrome, and elevated levels of fasting blood glucose, triglycerides and ALT enzyme. Regarding chronic diseases, when analyzing the conditions separately, the association of processed meat consumption with diabetes and hypertension appear influenced by obesity, while the association with cardiovascular disease was still evident when evaluating the obese and non-obese subgroups. To our knowledge, this is the first population-based cohort study to address associations between processed meat consumption and sociodemographic, lifestyle and health factors in Chile. The Latin American Study of Nutrition and Health (ELANS), conducted in 2014–2015 using 24-h recall in eight Latin American countries (Argentina, Brazil, Chile, Colombia, Costa Rica, Ecuador, Peru, and Venezuela), found that Chile had the highest processed meat consumption; in the region, processed meat intake was higher among men and showed a trend toward higher consumption at low socioeconomic status (28). Processed meat intake was also assessed in Chile in 2010 by a nationwide dietary survey (population ≥2 years of age) (33); geographical distribution was reported, with lower processed meat consumption in the north and higher consumption in the central (which included Maule Region, where the MAUCO cohort is located) and southern zones.

### Interpretation of findings

According to the last Chilean dietary survey (2010) (33), the population had a median processed meat consumption of 26.4 g/day; intake among respondents with similar characteristics to our participants in terms of age, sex, region, and rurality, ranged from 19 to 32 g/day. However, these figures are 10 years old, and since then the consumption of animal protein has increased in Chile (51). In our study, more than 25% of participants consumed processed meat twice or more per week. Translating portions to grams per day, we conservatively estimate that our participants in the highest intake category (≥5 times per week) are likely eating processed meat in the upper limit reported in 2010, i.e., 32 g/day. This is 10 times the daily amount of 0–4 g/day suggested to reduce the risk of chronic disease mortality and morbidity (2), including cancer (7). Although specific data on processed meat intake for the Maule Region is not available, Maule is the second largest processed meats producer in Chile (52), and is located in the Central macrozone which has one of the highest processed meat consumptions in the country (33). Interestingly, the lowest intake is in the Northern macrozone of Chile, which has lower cancer and cardiovascular disease mortality than the Central macrozone (53).

In MAUCO, 33% of participants reported being non-consumers of processed meat. This is similar to what was reported in Swiss population in 2014–2015 (54) and lower than what was reported for the countries in ELANS study, including Chile where 40% of the sample reported not consuming processed meat (28). Men ate more processed meat than women in MAUCO, which is consistent with previous reports in Chile (33), and in other populations in Latin America (28), Europe (3, 54–56), Australia (57) and the US (58). Younger people ate more processed meat, also previously reported in Chile (28, 33), other Latin American countries (28, 59), the US (56, 60), and Europe (61), while studies in Switzerland and Ireland found opposite trends (54, 62). However, it should be noted that given the age range of MAUCO participants (38–74 years), it was not possible to assess consumption in younger segments of the adult population. We did not find an association of processed meat consumption with schooling, a marker of socioeconomic status in Chile; this could be explained by its low variability in our sample, with an interquartile range of 6. On the other hand, being employed, a marker of higher current income, was associated with higher processed meat consumption. The association between socioeconomic status and processed meat intake is not clear in the international literature: In some high-income European countries, lower socio-economic groups are higher processed meat consumers (29, 61); in Switzerland, lower intake of processed meat was associated with higher education but not with income (54); in Latin America, although a higher consumption at lower socioeconomic status seems to be the current trend (28), older reports from Chile and Colombia show the opposite (33, 63). Overall, global meat consumption is rising (64) and processed meat consumption is particularly high with respect to an optimal intake (2), and is increasing in some low- and middle-income countries (29), even regardless of per capita family income in countries like Brazil (59).

Meat in general is a dietary source of several micronutrients, so a modest intake can be important for health and disease prevention (65). In the case of processed meat, however, no level of intake can confidently be associated with a lack of risk according to WCRF (1), especially in relation to cancer. Our findings of processed meat consumption being associated with poorer health are in accordance with the literature. Micha et al. (5) reported 42% higher risk of coronary heart disease and 19% higher risk of diabetes per 50 g/day. Chen et al. reported 11% higher risk of stroke per 50 g/day (7). In the US, processed meat was associated with incident cardiovascular disease (66). A recent meta-analysis of sixteen studies covering 10 countries reported that high consumers of processed meat had 35% higher risk of metabolic syndrome (20).

We found an association between processed meat consumption and obesity based on waist circumference or BMI, which is consistent with reports in Chile and elsewhere (55, 67–70). Additionally, we found an association of processed meat consumption with diabetes and hypertension, however, when stratifying by obesity (presence or absence) this association was not observed. On the other hand, a higher prevalence of cardiovascular disease was observed for high consumers, regardless of obesity status. We then confirm that the association of processed meat with diabetes was partly mediated by obesity (13.9%), as were cardiovascular disease and digestive symptoms, but to a lesser degree. A previous study in Chilean population reported that fatty and processed meats (≥1 vs. <1 portion/week) were associated with abdominal obesity (OR 1.30), and with metabolic syndrome components, including high blood glucose (OR 1.41) and high triglycerides (OR 1.19) (68). The association of processed meat intake with diabetes, partly mediated by obesity. has been reported in other populations (16, 17).

We also found a positive trend between processed meat consumption and elevated ALT enzyme (>55 UI/L) and fatty liver, the latter concordant with previous reports (71). The prospective cohort Nurses' Health Study II, with 77,795 women, reported that red meat consumption -unprocessed and processed- was associated with increased risk of non-alcoholic fatty liver disease (72).

In MAUCO, current smoking and binge drinking had a positive trend across processed meat categories. This has also been reported in European and Asian studies (62, 73, 74), while a meta-analysis including four studies in Europe and the US found an association of processed meat with current smoking but not with alcohol drinking (75). Another lifestyle behavior that has been related with high processed meat intake is low physical activity, with studies showing evidence of association in Switzerland (54), France (55) and Spain (70). In MAUCO, the prevalence of low physical activity (<30 min of physical activity 3 times/week), although high (>90%), was very similar across the five categories of intake (data not shown).

Higher amounts of processed meat are consumed in low quality diets, usually classified as western-type patterns and associated with several chronic conditions (76–78). Moreover, some processed meat products such as pre-made hamburgers, sausages and ham, are considered ultra-processed foods (79), a category that also includes carbonated soft drinks, chocolate, pastries, confectionery, mass-produced packaged breads, and margarines, among others, and that has already been associated with chronic conditions like cancer (80) and hypertension (73). In our study, a high intake of red meat, butter or cream, sugary snacks and sweets, sugary drinks, sugar, fried foods and chili peppers were associated with higher frequency of processed meat consumption. These findings are consistent with reports from the EPIC cohort (3) and NHANES III (81). Sugary drinks and sugars have also been associated with processed meat intake and unhealthy dietary patterns (62, 69, 82), making it challenging to identify the risk attributed to each food item. In MAUCO, chili pepper consumption was associated with higher processed meat consumption, as reported in the US population, where consumers of hot red chili pepper were more likely to be younger, male, to smoke cigarettes, drink alcohol, and consume meat (83). Nevertheless, a regular consumption of chili peppers appears to be more related to Mediterranean dietary patterns rather than to western types (84).

Although an inverse relation between processed meat and healthy foods is commonly reported (55, 62, 69), we observed this only for vegetables, and not for legumes, fish, seafood or avocados. This unexpected finding could be partially explained by the fact that in Chile men have a higher intake of legumes than women (28, 85), particularly in the Maule Region, which has the highest compliance with legume national recommendations (86) (≥2 times per week) (85). Additionally, although current recommendations advise against mixing legumes with processed meats, this is one of the most popular ways of consuming them in the country. The positive association of processed meat with fish and avocado in our study could be related to the higher price of these food items, considering that high processed meat consumers were more likely to be employed.

### Nutritional relevance of the findings and potential health impacts

Despite the fact that MAUCO participants are from a population with particularities in terms of location, exposures and sociodemographic changes, they have also been impacted by the so-called nutrition transition affecting the entire country. Although the direction of causation cannot be established in this study, the associations of higher processed meat consumption and chronic health conditions are in line with the international evidence, suggesting that a high consumption could promote obesity and associated diseases. However, due to the cross-sectional nature of the study design and the potential confounding role of other dietary factors (69), the results should be interpreted carefully. The findings of this study contribute to a better understanding of other relevant factors that go along with the consumption of processed meat in this population, as well as a better comprehension of this exposure in Chile. This will be useful information for future regulation efforts.

### Strengths and limitations of the study

MAUCO is a Chilean cohort with a comprehensive and detailed measurement collection. At baseline, participants answered health and risk factor surveys (exploring diet, alcohol, physical activity and health history, among others) including adapted nationally and internationally validated instruments. MAUCO constitutes an opportunity to address specific health needs of Chile's population in the context of accelerated development and nutritional transition (87); hence, the information obtained from this study will also be relevant for other Latin American populations. The food frequency questionnaire used for the dietary assessment was elaborated from a Mediterranean index (Chilean-MDI) with the advantage of being adapted and validated specifically for use in Chilean population (45).

Among the limitations of this study is that MAUCO is located in a Chilean agricultural county similar to the majority of small counties in the country but some results may not be applicable to residents of large urban areas in Chile (43). In addition, as the main objective of MAUCO is to study the natural history of chronic diseases in adult population from 38 years of age, the representativeness of the results in terms of processed meat intake is limited, as younger segments of the adult population were not included. With respect to diet, processed meat consumption was obtained in terms of weekly frequency, which is often accompanied with serving size estimations to have a better measurement of intake (88); we did not directly assess serving size, but we estimated it based on national nutrition surveys. Finally, being a cross-sectional analysis, it is not possible to establish causal relationship and reverse causality cannot be ruled out.

Future directions of this study include prospectively evaluating the association of processed meat consumption with incidence of chronic conditions, and identifying mediators or potentiators of the damage.

In conclusion, in this population, in addition to male sex and lower age, high processed meat intake was associated with other foods consumed at frequencies considered unhealthy, and with risky alcohol intake, unhealthy weight, and chronic diseases, particularly cardiovascular disease. However, no association was found between self-reported cancer and processed meat. Since this cohort resides in a region with a high incidence rate for gastric cancer and one of the highest mortality rates for colon cancer, future prospective studies are warranted in order to assess this association.

## Data availability statement

The datasets presented in this article are not readily available because they are available from the corresponding author upon reasonable request. Requests to access the datasets should be directed to CF, cferrec@med.puc.cl.

## Ethics statement

The studies involving human participants were reviewed and approved by the Ethics Committee of the School of Medicine at Pontificia Universidad Católica de Chile on 21 March 2019 (project 181010022). In addition, the MAUCO study protocol was approved by Ethics Committees at Pontificia Universidad Católica de Chile and the Maule Regional Service of the Chilean Ministry of Health. The patients/participants provided their written informed consent to participate in this study.

## Author contributions

JR: formal analysis, methodology, funding acquisition, investigation, and writing the manuscript. VC: data curation, formal analysis, and methodology. AH and VV: funding acquisition, investigation, and review & editing the manuscript. CV: formal analysis and methodology. CF: formal analysis, methodology, funding acquisition, investigation, supervision, review, and editing the manuscript. All authors contributed to the article and approved the submitted version.

## Funding

This research was funded by Fondo Nacional de Desarrollo Científico y Tecnológico (FONDECYT Postdoctoral), Grant Number 3190842 and Fondo de Financiamiento de Centros de Investigación en Áreas Prioritarias (FONDAP) (Grant Number 15130011).

## Conflict of interest

The authors declare that the research was conducted in the absence of any commercial or financial relationships that could be construed as a potential conflict of interest.

## Publisher's note

All claims expressed in this article are solely those of the authors and do not necessarily represent those of their affiliated organizations, or those of the publisher, the editors and the reviewers. Any product that may be evaluated in this article, or claim that may be made by its manufacturer, is not guaranteed or endorsed by the publisher.
