# Supplementary material for: Processed meat consumption and associated factors in Chile: A cross-sectional study nested in the MAUCO cohort
Source: Front Public Health. 2022 Aug 18;10:960997. doi: 10.3389/fpubh.2022.960997 (PMC9436317; doi:10.3389/fpubh.2022.960997)
Supplement: Supplementary file 1 [file Data_Sheet_1.docx]

Supplementary Material

**Table S1.** Selection of variables for multinomial model after multiple imputation. Analysis in 7,841 MAUCO participants.

| **Sociodemographic, Lifestyle**  **and Dietary variables** | **Models in which the variable**  **remained (%) *** |
| --- | --- |
| Sex ^†^ | 100 |
| Age ^†^ | 100 |
| Schooling ^†^ | 100 |
| Health Insurance | 100 |
| Employment status ^†^ | 100 |
| Drinking pattern | 100 |
| Legumes | 100 |
| Nuts | 100 |
| Red meat ^†^ | 100 |
| Fish/seafood | 100 |
| Butter/cream | 100 |
| Sugary snacks/sweets | 100 |
| Sugary drinks | 100 |
| Fresh green chili pepper | 100 |
| Dried red chili pepper | 100 |
| Fried foods | 100 |
| Avocados | 100 |
| Vegetables | 99 |
| Whole-fat dairy products | 94 |
| White meat | 93 |
| Skimmed/fermented dairy products | 66 |
| Fresh red chili pepper | 48 |
| Whole grain cereals | 4 |
| Smoking | 0 |
| Fruits | 0 |
| Olive oil | 0 |
| Sugar | 0 |

^†^ Adjustment variables; * Variables that remained in at least 60% of the models performed in the 100 imputed datasets.

**Table S2.** Missing data analysis, relationships between variables (rows) and missingness in variables (columns).

| **Missingness in variables  (% Missing)** | **Schooling** (5.4) | **Health Insurance** (3.8) | **Employment status** (20.4) | **Drinking pattern** (0.1) | **Legumes** (0.3) | **Nuts** (1.9) | **Red meat** (8.0) | **Fish / seafood** (0.5) | **Butter/cream** (1.5) | **Sugary snacks /sweets**  (1.7) | **Sugary drinks** (1.1) | **FGC pepper** (2.8) |
| --- | --- | --- | --- | --- | --- | --- | --- | --- | --- | --- | --- | --- |
| **Variables** |  |  |  |  |  |  |  |  |  |  |  |  |
| Processed meat | R |  | R |  |  | R | R | R | R | R | R | R |
| Sex | R |  | R | R |  |  | R |  |  |  |  |  |
| Age |  | R | R |  |  |  |  |  | R |  | R |  |
| Schooling | - | R | R |  |  | R |  |  |  |  |  |  |
| Health Insurance |  | - |  |  |  |  |  |  |  |  |  |  |
| Employment status | R | R | - |  |  | R | R |  | R |  |  | R |
| Drinking pattern | R |  | R | - |  |  | R |  |  |  | R |  |
| Legumes | R |  | R |  | - | R |  |  | R |  |  |  |
| Nuts | R | R |  |  |  | - |  |  |  |  | R |  |
| Red meat |  |  | R |  |  | R | - |  | R |  | R | R |
| Fish/seafood |  |  |  |  |  |  | R | - |  |  |  |  |
| Butter/cream | R | R | R |  |  | R |  | R | - |  | R | R |
| Sugary snacks/sweets |  |  | R |  |  |  | R |  |  | - |  | R |
| Sugary drinks | R |  | R |  |  | R | R | R | R | R | - |  |
| FGC pepper | R |  |  |  |  | R |  | R | R | R | R | - |
| DRC pepper | R | R |  |  |  | R | R |  | R | R | R | R |
| Fried foods |  | R | R |  |  |  | R |  |  |  |  |  |
| Avocados |  | R |  |  |  |  | R |  |  |  |  |  |
| Vegetables |  | R | R |  |  |  |  |  |  |  | R |  |
| Whole-fat dairy products |  |  | R |  |  | R |  |  | R | R | R | R |
| White meat |  |  |  |  |  |  |  |  |  | R |  |  |
| Skimmed/fermented dairy products | R |  | R |  |  |  |  |  | R |  | R | R |
| FRC pepper | R |  |  |  |  | R |  | R | R | R | R | R |
| Whole grain cereals |  |  | R |  |  |  |  |  |  | R | R | R |
| Smoking | R |  | R |  |  |  | R |  |  |  |  |  |
| Fruits |  | R | R |  | R |  |  |  |  |  |  |  |
| Olive oil |  |  |  |  | R |  |  |  |  |  |  |  |
| Sugar | R | R | R |  |  |  | R |  | R |  |  | R |

**Table S2.** Continued.

| **Missingness in variables  (% Missing)** | **DRC pepper** (3.0) | **Fried foods** (0.5) | **Avocados** (0.7) | **Vegetables** (0.4) | **Whole-fat dairy products** (2.2) | **White meat** (0.2) | **Skimmed/fermented dairy products** (2.1) | **FRC pepper** (3.4) | **Whole grain cereals** (4.3) | **Smoking** (0.2) | **Fruits** (0.1) | **Olive oil**  (3.5) | **Sugar** (2.2) |
| --- | --- | --- | --- | --- | --- | --- | --- | --- | --- | --- | --- | --- | --- |
| **Variables** |  |  |  |  |  |  |  |  |  |  |  |  |  |
| Processed meat | R |  | R |  | R |  | R | R | R |  |  | R | R |
| Sex |  |  |  |  |  |  |  | R |  | R |  |  |  |
| Age | R |  | R |  |  |  |  | R |  |  |  |  | R |
| Schooling |  |  |  |  |  |  |  |  |  |  |  |  |  |
| Health Insurance |  |  |  |  |  |  |  |  |  |  |  |  |  |
| Employment status | R |  |  |  | R |  | R | R | R |  | R | R | R |
| Drinking pattern |  |  |  |  |  |  |  |  |  |  |  |  |  |
| Legumes |  |  |  |  |  |  | R | R | R |  |  | R | R |
| Nuts |  |  |  |  |  |  |  |  |  |  |  |  |  |
| Red meat | R |  | R |  | R |  | R | R | R |  |  | R | R |
| Fish/seafood |  |  |  |  |  |  | R |  |  |  |  |  |  |
| Butter/cream | R |  |  |  | R |  | R | R | R |  |  |  | R |
| Sugary snacks/sweets |  |  |  |  |  |  |  |  |  |  |  |  |  |
| Sugary drinks | R | R |  |  | R |  | R | R | R |  |  | R |  |
| FGC pepper | R |  |  |  | R |  | R | R | R |  |  | R | R |
| DRC pepper | - |  |  | R | R |  | R |  | R |  |  | R | R |
| Fried foods |  | - |  |  | R |  |  |  |  |  |  |  |  |
| Avocados |  |  | - |  |  |  |  |  |  |  |  |  |  |
| Vegetables |  | R |  | - | R |  |  | R |  |  |  |  | R |
| Whole-fat dairy products | R |  |  |  | - |  | R | R | R |  |  | R | R |
| White meat |  |  |  |  |  | - |  |  |  |  |  |  |  |
| Skimmed/fermented dairy products | R |  |  |  | R |  | - | R | R |  |  | R | R |
| FRC pepper | R |  |  |  | R |  | R | - | R |  |  | R | R |
| Whole grain cereals | R |  |  |  |  |  |  | R | - |  |  | R | R |
| Smoking |  |  |  |  |  |  |  |  |  | - |  |  | R |
| Fruits |  |  |  | R |  |  |  |  |  |  | - |  |  |
| Olive oil |  |  |  | R |  |  |  |  |  |  |  | - |  |
| Sugar | R | R |  |  | R |  | R | R |  |  |  | R | - |

R: A relationship exist between the variable in row and the missingness in the variable in column, confirmed with chi square test for categorical or Kruskall Wallis test for continuous variables, using alpha ≤0.05. FGC: Fresh green chili; DRC: dried red chili; FRC: Fresh red chili. Only variables considered for multinomial model using MICE (in Table 5) were included.

**Table S3**. Number of participants with data and proportion with missing data for each variable.

| **Variables** | **Participants with data** (total= 7,841) | **Missing (%)** |
| --- | --- | --- |
| **Sociodemographic** |  |  |
| Sex | 7841 | 0.0 |
| Age | 7841 | 0.0 |
| Self-identified ethnicity | 7822 | 0.2 |
| Schooling | 7416 | 5.4 |
| Health insurance | 7545 | 3.8 |
| Employment status | 6243 | 20.4 |
| **Lifestyle & Diet** |  |  |
| Smoking | 7828 | 0.2 |
| Drinking pattern | 7833 | 0.1 |
| Vegetables | 7806 | 0.4 |
| Fruits | 7835 | 0.1 |
| Legumes | 7815 | 0.3 |
| Nuts | 7691 | 1.9 |
| Whole grain cereals | 7502 | 4.3 |
| White meat | 7825 | 0.2 |
| Processed meat | 7841 | 0.0 |
| Red meat | 7210 | 8.0 |
| Fish or seafood | 7798 | 0.5 |
| Skimmed/fermented dairy products | 7676 | 2.1 |
| Whole-fat dairy products | 7670 | 2.2 |
| Butter or cream | 7723 | 1.5 |
| Olive oil | 7564 | 3.5 |
| Avocados | 7788 | 0.7 |
| Sugary snacks/sweets | 7710 | 1.7 |
| Sugary drinks | 7755 | 1.1 |
| Sugar | 7670 | 2.2 |
| Fresh green chili pepper | 7622 | 2.8 |
| Fresh red chili pepper | 7577 | 3.4 |
| Dried red chili pepper | 7608 | 3.0 |
| Fried foods | 7805 | 0.5 |
| **Health** |  |  |
| Chronic conditions | 5534 | 29.4 |
| Remaining teeth | 7841 | 0.0 |
| Waist circumference | 7567 | 3.5 |
| Body mass index | 7427 | 5.3 |
| **Hepatobiliary ultrasound** |  |  |
| Fatty liver | 7402 | 5.6 |
| Gallbladder disease | 7258 | 7.4 |
| **Laboratory exams** |  |  |
| Fasting blood glucose | 7405 | 5.6 |
| LDL | 7256 | 7.5 |
| Triglycerides | 7396 | 5.7 |
| HDL | 7396 | 5.7 |
| AST | 7397 | 5.7 |
| ALT | 7395 | 5.7 |
| Metabolic Syndrome | 7405 | 5.6 |
